# Decreasing rates of major lower-extremity amputation in people with diabetes but not in those without: a nationwide study in Belgium

**DOI:** 10.1007/s00125-018-4655-6

**Published:** 2018-06-16

**Authors:** Heiner Claessen, Herve Avalosse, Joeri Guillaume, Maria Narres, Tatjana Kvitkina, Werner Arend, Stephan Morbach, Patrick Lauwers, Frank Nobels, Jacques Boly, Chris Van Hul, Kris Doggen, Isabelle Dumont, Patricia Felix, Kristien Van Acker, Andrea Icks

**Affiliations:** 1 Institute for Health Services Research and Health Economics, German Diabetes Center (DDZ), Leibniz Institute for Diabetes Research at Heinrich Heine University Düsseldorf, Auf’m Hennekamp 65, 40225 Düsseldorf, Germany; 2 Institute for Health Services Research and Health Economics, Faculty of Medicine, Heinrich Heine University Düsseldorf, Düsseldorf, Germany; 3 German Center for Diabetes Research (DZD), Munich-Neuherberg, Germany; 4 IMA/AIM (InterMutualistisch Agentschap/Agence Intermutualiste), Brussels, Belgium; 5 Landsbond der Christelijke Mutualiteiten/Alliance Nationale des Mutualités Chrétiennes, Brussels, Belgium; 6 Nationaal Verbond der Socialistische Mutualiteiten/Union Nationale des Mutualités Socialistes, Brussels, Belgium; 7 Department of Diabetes and Angiology, Marienkrankenhaus, Soest, Germany; 8 Diabetes Liga, Ghent, Belgium; 9 Department of Thoracic and Vascular Surgery, Antwerp University Hospital, Edegem, Belgium; 10 Department of Endocrinology, Onze Lieve Vrouw Ziekenhuis Aalst, Aalst, Belgium; 11 Landsbond van Onafhankelijke Ziekenfondsen/Union des Mutualités Libres, Anderlecht, Belgium; 12 Scientific Institute of Public Health, Brussels, Belgium; 13 Association Belge du Diabète, Brussels, Belgium; 14 Centre Multidisciplinaire du Pied Diabetique, Ransart, Belgium; 15 Department of Endocrinology, CHR de la Citadelle, Liège, Belgium; 16 D-Foot International (International Working Group on the Diabetic Foot- Implementation), Department of Diabetology and Endocrinology, CSF, Chimay, Belgium

**Keywords:** Amputation rate, Diabetes, Major lower-extremity amputation, Minor lower-extremity amputation, National health insurance funds

## Abstract

**Aims/hypothesis:**

The reduction of major lower-extremity amputations (LEAs) is one of the main goals in diabetes care. Our aim was to estimate annual LEA rates in individuals with and without diabetes in Belgium, and corresponding time trends.

**Methods:**

Data for 2009–2013 were provided by the Belgian national health insurance funds, covering more than 99% of the Belgian population (about 11 million people). We estimated the age–sex standardised annual amputation rate (first per year) in the populations with and without diabetes for major and minor LEAs, and the corresponding relative risks. To test for time trends, Poisson regression models were fitted.

**Results:**

A total of 5438 individuals (52.1% with diabetes) underwent a major LEA, 2884 people with above- and 3070 with below-the-knee major amputations. A significant decline in the major amputation rate was observed in people with diabetes (2009: 42.3; 2013: 29.9 per 100,000 person-years, 8% annual reduction, *p* < 0.001), which was particularly evident for major amputations above the knee. The annual major amputation rate remained stable in individuals without diabetes (2009: 6.1 per 100,000 person-years; 2013: 6.0 per 100,000 person-years, *p* = 0.324) and thus the relative risk reduced from 6.9 to 5.0 (*p* < 0.001). A significant but weaker decrease was observed for minor amputation in individuals with and without diabetes (5% and 3% annual reduction, respectively, *p* < 0.001).

**Conclusions/interpretation:**

In this nationwide study, the risk of undergoing a major LEA in Belgium gradually declined for individuals with diabetes between 2009 and 2013. However, continued efforts should be made to further reduce the number of unnecessary amputations.

**Electronic supplementary material:**

The online version of this article (10.1007/s00125-018-4655-6) contains peer-reviewed but unedited supplementary material, which is available to authorised users.

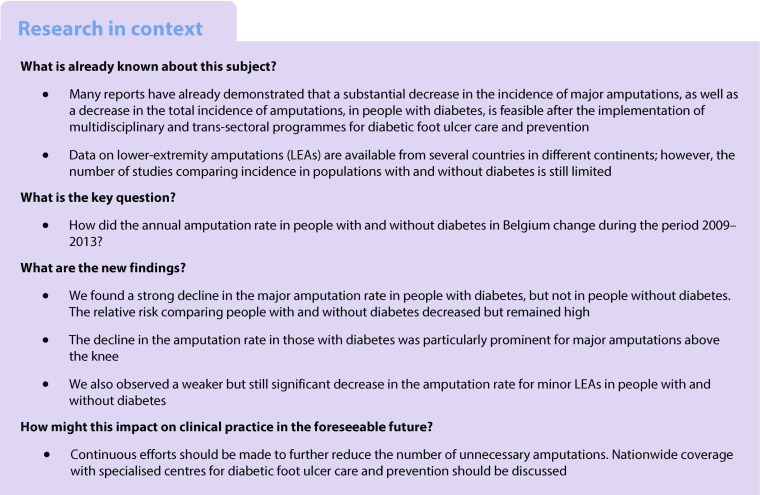



## Introduction

Lower-extremity amputations (LEAs) have a huge impact on individuals and also on society [1, 2]. Practical issues such as reduced mobility, pain, hospitalisation, revalidation, disability and unemployment, a changed self-image and difficulties with activities of daily living lead to reduced quality of life for the person affected and their relatives. There are also considerable financial consequences—especially with a major amputation [1].

A substantial proportion of LEAs, particularly in people with diabetes, are thought to be preventable via the provision of appropriate healthcare. Many reports have already demonstrated that a substantial decrease in the incidence of major amputations, as well as a decrease in the total incidence of amputations, in people with diabetes, is feasible after implementation of a multidisciplinary programme for the prevention and treatment of diabetic foot ulcers, including earlier and more frequent use of revascularisation procedures [3, 4].

Such a programme to prevent and treat the diabetic foot ulcer has been introduced in Belgium. In 1989, the first multidisciplinary foot clinic was established at the University of Antwerp, followed by a gradual process of decentralisation. In the early 1990s, national campaigns were organised at the primary care level. In 2005, a national diabetic foot care programme was established involving recognised diabetic foot clinics. The number of diabetic foot clinics recognised by the Belgian Ministry of Health increased from 21 in 2008 to 34 in 2014. In order to maintain this recognition, diabetic foot clinics are required to participate in a quality-improvement initiative (Initiative for Quality Improvement and Epidemiology in Multidisciplinary Diabetic Foot Clinics [IQED-Foot]). The activities within this initiative can be summarised as follows: evaluating quality of care based on repeated audits; improving quality of care by providing individual feedback with anonymous benchmarking; and organising informative meetings to add to scientific knowledge on the presentation and management of and outcomes in people with diabetic foot ulcers [5, 6].

Information on the incidence of LEAs that is accurate, up-to-date and comparable is essential to guide and monitor interventions aimed at LEA prevention [3, 7]. Data on LEAs are available from several countries in different continents. However, the number of studies that have estimated the risk of amputation among the population ‘at risk’ (i.e. in the populations with and without diabetes, respectively) remains limited [8–15]. In order to evaluate which changes are specific for the diabetic situation, knowledge is needed both of the incidence of LEAs in the non-diabetic population and of RRs.

Hence, the aim of this study was to analyse the annual major as well as minor LEA rates in people with and without diabetes in Belgium, and to evaluate whether these changed during the period 2009–2013. To the best of our knowledge, this paper is the first to evaluate the LEA rate in Belgium based on a nationwide dataset.

## Methods

### Study population and data assessment

The study population comprised almost the entire Belgian population (>99%). We used data from the Belgian national health insurance funds, provided by the InterMutualistisch Agentschap/Agence InterMutualiste (IMA/AIM), for all individuals who were insured for at least one day in Belgium between 1 January 2009 and 31 December 2013. The following information was available for all insured people: year of birth (based on 5 year intervals), sex, diagnosis of diabetes and amputation (including level of amputation).

Individuals were classified as ‘with diabetes’ if they met at least one of the following criteria: (1) inclusion in a diabetes care programme; (2) treatment with diabetes-specific medication; and/or (3) registration of repeated HbA_1c_ measurements. Inclusion in one of the three diabetes care programmes in Belgium could be defined by ownership of a ‘diabetes passport’, inclusion in a diabetes care plan, inclusion in a diabetes meeting or patient reimbursement of glucose meters, strips and lancets. Treatment with diabetes-specific medication was defined as intake of glucose-lowering medication based on the World Health Organization classification within one calendar year (ATC codes A10A and A10B, at least 90 defined daily doses per year). Registration for repeated measurement of HbA_1c_ levels was considered if at least three HbA_1c_ measurements had been carried out over two consecutive calendar years. Hospitalised individuals who had drugs issued only by the hospital pharmacy were excluded because this could be related to transient hyperglycaemia during acute illness in people otherwise not suffering from diabetes. Likewise, gestational diabetes, which was ascertained when a woman received glucose-lowering medication only during pregnancy, was excluded.

LEAs were classified according to the official nomenclature codes (provided by the IMA/AIM). We further differentiated between major amputation (any amputation above the ankle) and minor amputation (below the ankle); major LEAs were additionally subdivided into major below-the-knee amputation and major above-the-knee LEA (knee disarticulation or proximal).

These data were anonymised, aggregated and analysed by blinded investigators. Therefore, neither ethical approval nor individual written consent from participants was required.

### Statistical analysis

We conducted all analyses for the entire population, and stratified by sex. They were performed for major LEAs, major LEAs above and below the knee, and minor LEAs as outcomes. Furthermore, all LEAs were additionally analysed.

We computed diabetes prevalence and assessed the time trend using the *χ*^2^ test. For each outcome, we estimated the amputation rate for each calendar year as follows: the number of people with an outcome occurring within this year as numerator was divided by the number of insured people in the respective year as denominator. Therefore, the amputation rate could count one person several times in different calendar years if multiple outcomes occurred in different years (e.g. major amputation of the left leg in 2009, major amputation of the right leg in 2010). A major LEA was also counted for analysis if a person had previously undergone a minor LEA, even if this was in the same calendar year.

We directly computed age–sex standardised amputation rates and, for the sex-specific analyses, age–standardised amputation rates using 0–39, 40–49, 50–59, 60–69, 70–79, 80+ years as age strata (standard population: Belgian population 2011) for each calendar year for the estimated population with diabetes (amputation rate for the estimated population with diabetes [ARd]) and the population without diabetes (amputation rate for the population without diabetes [ARn]), respectively. For international comparisons, we also standardised the major amputation rate for the European Standard Population (ESP) 2013 as sensitivity analysis [16]. Furthermore, we calculated RR in order to divide the amputation rates in individuals with diabetes by those in people without diabetes (ARd/ARn).

In order to test for time trends, we fitted separate Poisson regression models for the population with and without diabetes using year of outcome (difference from the first year 2009 as an ordinal variable), age (groups as described above) and sex as independent variables. Additionally, we calculated Poisson regression models for the entire population. In these, an additional variable for the presence of diabetes (‘yes’ vs ‘no’) was included, as well as an interaction term ‘presence of diabetes’ with ‘years since 2009’. All models were adjusted for over-dispersion using a dispersion parameter.

All analyses were conducted using the Statistical Analysis System (SAS for Windows 7, Release 9.4 TS1M2, SAS Institute, Cary, NC, USA).

## Results

### Study population

**Fig. 1:**
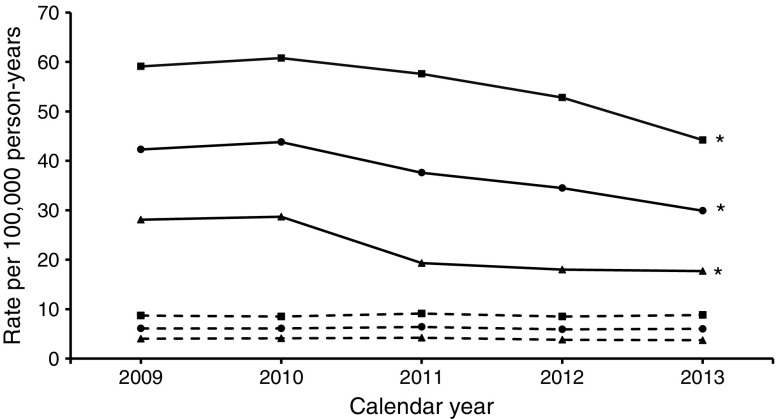
Time trend of age- and sex-standardised major amputation rate. Solid lines, people with diabetes; dashed lines, people without diabetes; circles, men and women; squares, men; triangles, women. *p < 0.05 for time trend (Poisson model)

**Fig. 2:**
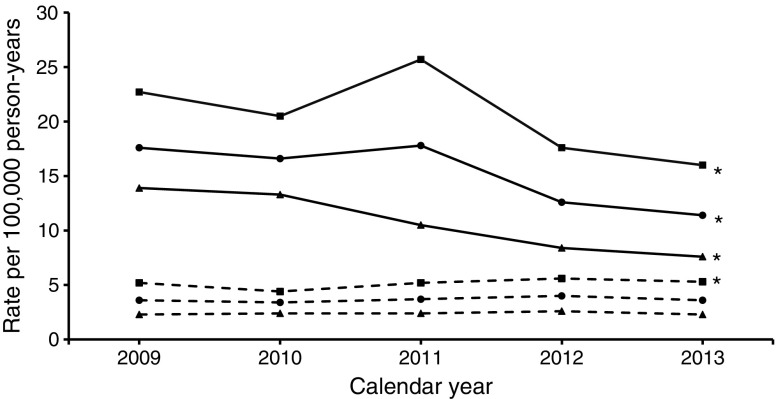
Time trend of age- and sex-standardised major above-the-knee amputation rate. Solid lines, people with diabetes; dashed lines, people without diabetes; circles, men and women; squares, men; triangles, women. *p < 0.05 for time trend (Poisson model)

**Fig. 3:**
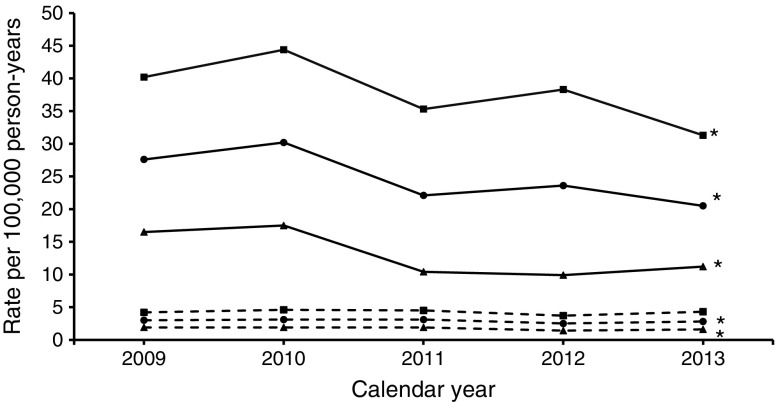
Time trend of age- and sex-standardised major below-the-knee amputation rate. Solid lines, people with diabetes; dashed lines, people without diabetes; circles, men and women; squares, men; triangles, women. *p < 0.05 for time trend (Poisson model)

**Fig. 4:**
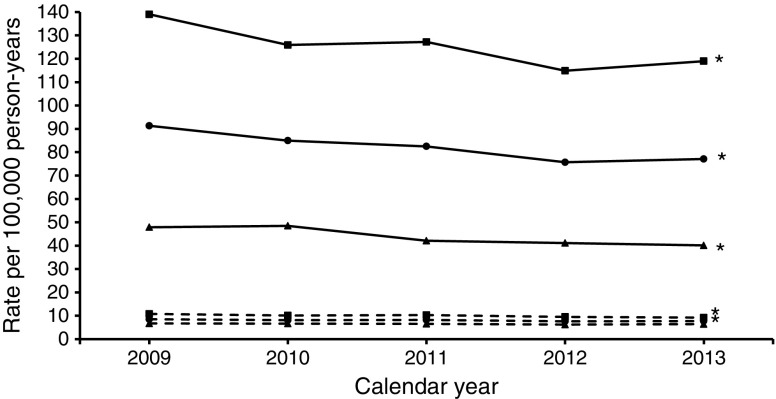
Time trend of age- and sex-standardised minor amputation rate. Solid lines, people with diabetes; dashed lines, people without diabetes; circles, men and women; squares, men; triangles, women. *p < 0.05 for time trend (Poisson model)

The description of the background population, numbers and age–sex standardised rates of major amputation (any major, major above the knee, major below the knee) and minor amputation, as well as corresponding RRs, are shown for each calendar year in Table 1 and Figs 1, 2, 3 and 4. The results of the time trend for the major and minor amputation rates in the population with diabetes from the fully adjusted Poisson models are presented in Table 2. The total study population comprised approximately 11 million insurants (2009: 10,877,318; 2013: 11,165,978). Diabetes prevalence rose from 6.2% in 2009 to 8.0% in 2013 (*χ*^2^ test *p* < 0.001), with somewhat higher values in the female population.

**Table 1:** Baseline characteristics of major and minor amputations, Belgium, 2009–2013

Population/calendar year	Diabetes				No diabetes				
	Number of people with amputation	Mean age^a^ (years)	Number of total population	ARd^b^	Number of people with amputation	Mean age^a^ (years)	Number of total population	ARn^b^	RR^c^
All major amputations									
Total population									
2009	618	72.4	678,655	42.3	554	69.5	10,198,663	6.1	6.9
2010	631	71.9	738,256	43.8	549	69.9	10,226,043	6.1	7.2
2011	590	71.9	793,883	37.6	578	69.9	10,255,320	6.4	5.9
2012	569	71.6	846,796	34.5	537	69.3	10,271,802	5.9	5.8
2013	599	71.9	896,126	29.9	542	69.7	10,269,852	6.0	5.0
Male population									
2009	404	71.0	326,803	59.1	347	66.2	5,021,923	8.7	6.8
2010	400	70.5	354,104	60.8	336	66.4	5,038,345	8.5	7.2
2011	399	70.0	379,168	57.6	360	67.7	5,056,551	9.1	6.3
2012	385	69.9	402,725	52.8	341	66.9	5,067,837	8.5	6.2
2013	411	70.8	424,291	44.2	351	67.5	5,068,800	8.8	5.0
Female population									
2009	214	75.1	351,852	28.1	207	75.1	5,176,740	4.0	7.1
2010	231	74.3	384,152	28.7	213	75.3	5,187,698	4.1	7.1
2011	191	75.7	414,715	19.3	218	73.7	5,198,769	4.2	4.6
2012	184	75.3	444,071	18	196	73.5	5,203,965	3.8	4.8
2013	188	74.3	471,835	17.7	191	73.8	5,201,052	3.7	4.8
Major amputations above the knee									
Total population									
2009	281	73.8	678,655	17.6	323	71.2	10,198,663	3.6	4.9
2010	285	74.4	738,256	16.6	300	72.3	10,226,043	3.4	4.9
2011	274	73.8	793,883	17.8	329	71.7	10,255,320	3.7	4.8
2012	259	74.4	846,796	12.6	356	71.0	10,271,802	4.0	3.2
2013	247	73.3	896,126	11.4	328	71.1	10,269,852	3.6	3.2
Male population									
2009	173	72.6	326,803	22.7	202	67.8	5,021,923	5.2	4.4
2010	164	72.7	354,104	20.5	173	69.0	5,038,345	4.4	4.6
2011	166	71.9	379,168	25.7	202	69.2	5,056,551	5.2	5.0
2012	161	72.1	402,725	17.6	221	68.7	5,067,837	5.6	3.1
2013	159	71.5	424,291	16.0	209	67.8	5,068,800	5.3	3.1
Female population									
2009	108	75.7	351,852	13.9	121	76.9	5,176,740	2.3	6.1
2010	121	76.7	384,152	13.3	127	76.6	5,187,698	2.4	5.5
2011	108	76.8	414,715	10.5	127	75.8	5,198,769	2.4	4.3
2012	98	78.2	444,071	8.4	135	74.8	5,203,965	2.6	3.2
2013	88	76.6	471,835	7.6	119	76.9	5,201,052	2.3	3.3
Major amputations below the knee									
Total population									
2009	385	71.3	678,655	27.6	271	67.2	10,198,663	3.0	9.3
2010	399	69.9	738,256	30.2	284	66.9	10,226,043	3.1	9.7
2011	360	70.2	793,883	22.1	281	67.9	10,255,320	3.1	7.2
2012	342	69.5	846,796	23.6	224	66.7	10,271,802	2.5	9.6
2013	393	70.6	896,126	20.5	256	68.0	10,269,852	2.8	7.3
Male population									
2009	262	69.9	326,803	40.2	174	64.0	5,021,923	4.2	9.5
2010	271	69.3	354,104	44.4	185	63.8	5,038,345	4.6	9.7
2011	264	68.9	379,168	35.3	180	65.9	5,056,551	4.5	7.9
2012	251	68.4	402,725	38.3	151	64.9	5,067,837	3.7	10.2
2013	282	70.0	424,291	31.3	173	67.3	5,068,800	4.3	7.3
Female population									
2009	123	74.2	351,852	16.5	97	72.9	5,176,740	1.9	8.9
2010	128	71.1	384,152	17.5	99	72.9	5,187,698	1.9	9.3
2011	96	73.8	414,715	10.4	101	71.4	5,198,769	1.9	5.4
2012	91	72.4	444,071	9.9	73	70.4	5,203,965	1.4	7.0
2013	111	72.0	471,835	11.2	83	69.4	5,201,052	1.6	7.1
Minor amputations									
Total population									
2009	1232	70.3	678,655	91.3	772	68.9	10,198,663	8.5	10.7
2010	1242	70.9	738,256	85.0	740	68.4	10,226,043	8.1	10.5
2011	1223	70.0	793,883	82.5	746	67.0	10,255,320	8.2	10.1
2012	1299	70.7	846,796	75.7	695	67.7	10,271,802	7.6	9.9
2013	1325	70.9	896,126	77.1	704	67.6	10,269,852	7.7	10.1
Male population									
2009	860	68.6	326,803	139.0	423	66.5	5,021,923	10.8	12.9
2010	838	69.3	354,104	125.9	397	65.5	5,038,345	10.1	12.5
2011	860	68.7	379,168	127.2	407	65.2	5,056,551	10.3	12.4
2012	903	69.6	402,725	114.9	373	66.0	5,067,837	9.5	12.1
2013	935	69.8	424,291	119.0	367	65.4	5,068,800	9.2	13
Female population									
2009	372	74.1	351,852	47.9	349	71.7	5,176,740	6.7	7.2
2010	404	74.3	384,152	48.5	343	71.7	5,187,698	6.6	7.4
2011	363	72.9	414,715	42.1	339	69.1	5,198,769	6.5	6.5
2012	396	73.3	444,071	41.1	322	69.8	5,203,965	6.2	6.6
2013	390	73.7	471,835	40.1	337	70.0	5,201,052	6.4	6.2

^a^Age at time of first amputation

^b^Amputation rate per 100,000 person-years in the population with diabetes and without diabetes, standardised to the Belgian population 2011

^c^RR comparing amputation rates in the population with and without diabetes (ARd/ARn)

**Table 2:** Results of Poisson models: relative risks for amputation, Belgium 2009–2013

Variables	RR (95% CI)		
	Total population	Men	Women
Any major amputation			
Model 1a (diabetes)			
Calendar year	0.920 (0.909, 0.931)*	0.933 (0.920, 0.946)*	0.894 (0.877, 0.911)*
Male vs female	2.258 (2.177, 2.342)*	–	–
Age (years)^a^			
≥80	18.824 (15.162, 23.369)*	10.618 (8.424, 13.383)*	52.671 (33.593, 82.583)*
70–79	13.688 (11.027, 16.991)*	8.418 (6.686, 10.598)*	34.452 (21.958, 54.056)*
60–69	9.308 (7.493, 11.563)*	6.100 (4.843, 7.682)*	19.087 (12.132, 30.029)*
50–59	6.759 (5.426, 8.419)*	4.161 (3.293, 5.258)*	16.967 (10.748, 26.786)*
40–49	3.39 (2.673, 4.299)*	1.857 (1.432, 2.409)*	10.543 (6.569, 16.924)*
Model 1b (no diabetes)			
Calendar year	0.991 (0.974, 1.009)	1.001 (0.981, 1.022)	0.975 (0.951, 1.000)
Male vs female	2.242 (2.130, 2.360)*	–	–
Age (years)^a^			
≥80	71.017 (63.367, 79.591)*	59.204 (51.699, 67.799)*	81.253 (68.314, 96.642)*
70–79	38.504 (34.279, 43.249)*	42.539 (37.225, 48.612)*	31.441 (26.184, 37.752)*
60–69	18.428 (16.345, 20.776)*	22.005 (19.210, 25.207)*	11.759 (9.650, 14.328)*
50–59	9.538 (8.417, 10.808)*	10.273 (8.908, 11.847)*	8.052 (6.589, 9.840)*
40–49	3.604 (3.111, 4.176)*	4.098 (3.477, 4.830)*	2.579 (2.002, 3.321)*
Model 2 (both diabetes and no diabetes combined)			
Calendar year	0.991 (0.974, 1.008)	1.001 (0.980, 1.021)	0.975 (0.950, 1.002)
Diabetes (yes vs no)	6.122 (5.776, 6.489)*	6.336 (5.908, 6.796)*	5.708 (5.211, 6.253)*
Male vs female	2.252 (2.174, 2.336)*	–	–
Age (years)^a^			
≥80	53.132 (47.956, 59.028)*	44.619 (39.564, 50.494)*	65.211 (55.092, 77.842)*
70–79	33.552 (30.274, 37.286)*	33.634 (29.871, 38.007)*	33.117 (27.880, 39.655)*
60–69	19.63 (17.692, 21.839)*	21.278 (18.890, 24.052)*	15.080 (12.608, 18.170)*
50–59	11.378 (10.218, 12.699)*	11.572 (10.231, 13.130)*	10.734 (8.933, 12.986)*
40–49	4.192 (3.692, 4.764)*	4.266 (3.690, 4.939)*	4.002 (3.214, 4.995)*
Diabetes × calendar year	0.927 (0.905, 0.949)*	0.932 (0.906, 0.958)*	0.916 (0.882, 0.951)*
Major amputation above the knee			
Model 1a (diabetes)			
Calendar year	0.902 (0.888, 0.917)*	0.914 (0.896, 0.933)*	0.883 (0.863, 0.905)*
Male vs female	1.869 (1.783, 1.959)*	–	–
Age (years)^a^			
≥80	39.803 (27.408, 57.803)*	23.887 (15.173, 37.606)*	74.895 (40.68, 137.884)*
70–79	24.376 (16.781, 35.41)*	15.969 (10.15, 25.125)*	41.965 (22.769, 77.345)*
60–69	14.385 (9.89, 20.922)*	10.324 (6.557, 16.256)*	19.257 (10.403, 35.644)*
50–59	9.124 (6.247, 13.326)*	6.197 (3.916, 9.805)*	14.368 (7.709, 26.779)*
40–49	4.681 (3.131, 6.998)*	2.342 (1.417, 3.87)*	11.422 (6.02, 21.671)*
Model 1b (no diabetes)			
Calendar year	1.018 (0.998, 1.039)	1.029 (1.004, 1.054)*	1.003 (0.974, 1.032)
Male vs female	2.155 (2.032, 2.285)*	–	–
Age (years)^a^			
≥80	110.327 (94.446, 128.879)*	93.062 (76.83, 112.723)*	120.183 (95.767, 150.824)*
70–79	61.266 (52.349, 71.702)*	69.182 (57.253, 83.596)*	48.389 (38.246, 61.223)*
60–69	27.25 (23.182, 32.031)*	33.959 (28.021, 41.155)*	16.077 (12.499, 20.681)*
50–59	12.64 (10.677, 14.965)*	14.959 (12.246, 18.273)*	8.535 (6.555, 11.113)*
40–49	4.55 (3.741, 5.533)*	5.241 (4.168, 6.591)*	3.282 (2.396, 4.497)*
Model 2 (both diabetes and no diabetes combined)			
Calendar year	1.018 (1.000, 1.037)	1.028 (1.005, 1.052)*	1.003 (0.976, 1.031)
Diabetes (yes vs no)	4.747 (4.441, 5.074)*	4.603 (4.233, 5.004)*	4.893 (4.433, 5.400)*
Male vs female	2.024 (1.946, 2.105)*	–	–
Age (years)^a^			
≥80	95.278 (83.499, 109.273)*	84.078 (71.588, 99.458)*	104.238 (85.069, 129.41)*
70–79	55.2 (48.34, 63.351)*	59.146 (50.414, 69.898)*	49.112 (39.935, 61.166)*
60–69	28.1 (24.56, 32.308)*	33.297 (28.351, 39.388)*	18.763 (15.119, 23.553)*
50–59	14.174 (12.324, 16.373)*	16.083 (13.617, 19.121)*	10.681 (8.521, 13.525)*
40–49	5.129 (4.36, 6.046)*	5.321 (4.382, 6.483)*	4.788 (3.692, 6.239)*
Diabetes × calendar year	0.886 (0.862, 0.910)*	0.889 (0.86, 0.92)*	0.881 (0.846, 0.918)*
Major amputation below the knee			
Model 1a (diabetes)			
Calendar year	0.926 (0.913, 0.938)*	0.941 (0.927, 0.956)*	0.889 (0.867, 0.911)*
Male vs female	2.649 (2.541, 2.762)*	–	–
Age (years)^a^			
≥80	12.335 (9.902, 15.367)*	7.638 (6.065, 9.619)*	35.56 (21.074, 60.004)*
70–79	11.139 (8.949, 13.864)*	7.057 (5.615, 8.869)*	31.227 (18.506, 52.692)*
60–69	8.525 (6.845, 10.616)*	5.647 (4.493, 7.097)*	20.585 (12.17, 34.817)*
50–59	6.427 (5.147, 8.026)*	3.99 (3.164, 5.031)*	19.349 (11.403, 32.83)*
40–49	3.042 (2.388, 3.875)*	1.795 (1.386, 2.324)*	9.989 (5.758, 17.328)*
Model 1b (no diabetes)			
Calendar year	0.964 (0.944, 0.984)*	0.977 (0.952, 1.002)	0.940 (0.910, 0.971)*
Male vs female	2.463 (2.315, 2.621)*	–	–
Age (years)^a^			
≥80	48.973 (43.346, 55.332)*	41.896 (36.022, 48.727)*	56.046 (46.219, 67.964)*
70–79	26.496 (23.376, 30.033)*	29.954 (25.832, 34.734)*	19.987 (16.21, 24.644)*
60–69	14.072 (12.373, 16.004)*	16.215 (13.947, 18.852)*	9.597 (7.682, 11.989)*
50–59	8.469 (7.422, 9.664)*	8.226 (7.025, 9.632)*	8.97 (7.235, 11.122)*
40–49	3.331 (2.851, 3.893)*	3.805 (3.182, 4.551)*	2.229 (1.671, 2.973)*
Model 2 (both diabetes and no diabetes combined)			
Calendar year	0.964 (0.944, 0.984)*	0.976 (0.952, 1.001)	0.941 (0.909, 0.973)*
Diabetes (yes vs no)	8.049 (7.533, 8.602)*	8.488 (7.835, 9.198)*	7.251 (6.491, 8.102)*
Male vs female	2.571 (2.469, 2.681)*	–	–
Age (years)^a^			
≥80	31.797 (28.487, 35.592)*	27.494 (24.137, 31.43)*	39.408 (32.537, 48.219)*
70–79	23.237 (20.83, 25.996)*	22.855 (20.123, 26.057)*	23.646 (19.455, 29.022)*
60–69	15.691 (14.06, 17.561)*	16.132 (14.207, 18.388)*	13.513 (11.058, 16.665)*
50–59	10.162 (9.078, 11.404)*	9.556 (8.383, 10.932)*	11.6 (9.476, 14.326)*
40–49	3.826 (3.346, 4.378)*	3.888 (3.333, 4.543)*	3.609 (2.812, 4.642)*
Diabetes × calendar year	0.960 (0.934, 0.986)*	0.963 (0.933, 0.995)*	0.944 (0.901, 0.989)*
Minor amputation			
Model 1a (diabetes)			
Calendar year	0.954 (0.945, 0.962)*	0.957 (0.948, 0.967)*	0.946 (0.932, 0.961)*
Male vs female	2.528 (2.458, 2.599)*	–	–
Age (years)^a^			
≥80	12.957 (11.216, 14.968)*	9.039 (7.666, 10.658)*	22.655 (17.640, 29.096)*
70–79	10.248 (8.874, 11.835)*	7.327 (6.221, 8.631)*	17.735 (13.805, 22.785)*
60–69	8.215 (7.112, 9.490)*	6.204 (5.267, 7.307)*	12.224 (9.498, 15.732)*
50–59	5.897 (5.095, 6.826)*	4.576 (3.878, 5.401)*	7.918 (6.117, 10.250)*
40–49	3.174 (2.709, 3.719)*	2.356 (1.968, 2.821)*	5.048 (3.829, 6.655)*
Model 1b (no diabetes)			
Calendar year	0.973 (0.959, 0.988)*	0.963 (0.944, 0.982)*	0.986 (0.965, 1.008)
Male vs female	1.541 (1.477, 1.608)*	–	–
Age (years)^a^			
≥80	40.665 (37.649, 43.924)*	42.094 (37.984, 46.65)*	37.438 (33.474, 41.872)*
70–79	19.135 (17.641, 20.756)*	20.454 (18.394, 22.744)*	17.29 (15.329, 19.503)*
60–69	8.984 (8.243, 9.792)*	10.976 (9.845, 12.238)*	6.586 (5.755, 7.537)*
50–59	4.66 (4.249, 5.110)*	5.298 (4.714, 5.954)*	3.843 (3.331, 4.434)*
40–49	1.944 (1.736, 2.177)*	2.143 (1.858, 2.471)*	1.683 (1.410, 2.010)*
Model 2 (both diabetes and no diabetes combined)			
Calendar year	0.973 (0.954, 0.992)*	0.962 (0.939, 0.987)*	0.986 (0.962, 1.010)
Diabetes (yes vs no)	9.457 (8.906, 10.045)*	11.955 (11.100, 12.882)*	6.448 (5.937, 7.003)*
Male vs female	2.088 (2.016, 2.165)*	–	–
Age (years)^a^			
≥80	25.967 (23.733, 28.462)*	23.172 (20.694, 26.017)*	28.283 (25.173, 31.882)*
70–79	16.864 (15.410, 18.488)*	15.823 (14.146, 17.747)*	17.48 (15.518, 19.753)*
60–69	11.615 (10.609, 12.739)*	12.019 (10.751, 13.473)*	9.399 (8.303, 10.669)*
50–59	6.896 (6.273, 7.592)*	7.448 (6.639, 8.376)*	5.241 (4.585, 6.003)*
40–49	2.731 (2.431, 3.068)*	2.894 (2.516, 3.33)*	2.392 (2.031, 2.816)*
Diabetes × calendar year	0.979 (0.956, 1.003)*	0.993 (0.964, 1.023)*	0.96 (0.928, 0.992)*

^a^All age groups are compared with the reference category, 0–39 years

*p < 0.05 vs reference category

### Major amputation

We identified 5438 individuals with any major amputation in the period 2009–2013, of whom almost two-thirds were male (65%). More than half (52%) of all individuals with an LEA were people with diabetes. The mean age of all amputees (71 years) remained nearly stable over the period. However, women were markedly older than men at the time of major amputation (75 vs 70 years) and the people with diabetes were older compared with people without diabetes (72 vs 70 years). These numbers remained nearly stable in the period 2009–2013. We identified 3070 individuals undergoing major amputation below the knee (men: 69%; diabetes: 59%) and 2884 individuals undergoing a major amputation above the knee (men: 61%; diabetes: 45%).

Throughout the observation period, the major amputation risk was more than six times higher in people with diabetes compared with individuals without diabetes (RR 6.122; 95% CI 5.776, 6.489) (Table 2). This difference was particularly evident when only amputations below the knee were considered (RR 8.049; 95% CI 7.533, 8.602) but also remained significantly increased for amputations above the knee (RR 4.747; 95% CI 4.441, 5.074).

We observed a significant decrease in the rate of any major amputation in the population with diabetes, from 42.3 per 100,000 person-years in 2009 to 29.9 in 2013 (Table 1, Fig. 1), with an annual reduction of 8% (RR per calendar year 0.920; 95% CI 0.909, 0.931) (Table 2). In contrast, no decline was observed in the population without diabetes (2009: 6.1; 2013: 6.0 [Table 1]; RR per calendar year 0.991; 95% CI 0.974, 1.009 [Table 2]). As a result, the RR comparing any major amputation rate in the population with and without diabetes decreased significantly by 7% per year (RR interaction diabetes × calendar year: 0.927; 95% CI 0.905, 0.949) (Table 2). The decrease in any major amputation rate in the population with diabetes was more prominent when considering solely major amputations above the knee (2009: 17.6; 2013: 11.4) (Table 1, Fig. 2) with an annual decline of 10% (RR per calendar year 0.902; 95% CI 0.888, 0.917) (Table 2). With regard to major amputations below the knee, we observed a weaker but still significant decrease (2009: 27.6; 2013: 20.5) (Table 1, Fig. 3) with an annual reduction of 7% (RR per calendar year 0.926; 95% CI 0.913, 0.938) (Table 2).

In the population without diabetes, the major above-the-knee amputation rate remained constant (2009: 3.6; 2013: 3.6) (Fig. 2) whereas there was a significant decrease in major below-the-knee amputations, with an annual reduction of 4% (RR 0.964; 95% CI 0.944, 0.984) (Table 2, Fig. 3). Thus, the reduction in the RR comparing the rates among people with and without diabetes was particularly strong when only amputations above the knee were taken into account (RR 0.886; 95% CI 0.862, 0.910) but also remained significant considering only amputations below the knee (RR 0.960; 95% CI 0.934, 0.986).

The major amputation rates were more than twice as high in men compared with women and strongly increased with age, which was true among people with, as well as those without, diabetes (Table 2). The reduction in the major amputation rate was more pronounced among women in all subgroups, while the change in RR was comparable in both sexes. The major amputation rate did not materially alter after standardisation for the ESP 2013 (electronic supplementary material [ESM] Table 1).

### Minor amputation

We identified 8811 people undergoing minor amputation (men: 62.8%; diabetes: 60.7%) (Table 1 and Fig. 4). We observed a significant decrease in the minor amputation rate in the population with diabetes from 91.3 per 100,000 person-years in 2009 to 77.1 in 2013 (5% annual reduction; RR per calendar year 0.954; 95% CI 0.945, 0.962; Table 2, Fig. 4). Likewise, a consistent decrease was seen among people without diabetes (2009: 8.5 per 100,000 person-years; 2013: 7.7; 3% annual reduction; RR per calendar year 0.973; 95% CI 0.959, 0.988), which was significant in men but not in women. Over the whole observation period, the RR of a minor LEA in the population with diabetes compared with the population without diabetes was 9.457 (8.906, 10.045). This relative risk did not change significantly between 2009 and 2013 (RR interaction diabetes × calendar year: 0.979; 0.956, 1.003; Table 2). The minor LEA AR was more than twofold higher in men compared with women. The decrease in minor LEA AR in the population with diabetes was more pronounced in women, while in the population without diabetes it was more prominent in men.

### Any amputation

In total, 12,899 people underwent any LEA (62% men, 56% with diabetes; mean age 70 years) (ESM Table 2). We observed a substantial decline in the any amputation rate (ESM Fig. 1) in the population with diabetes, from 122.2 per 100,000 person-years in 2009 to 100.4 in 2013, 5% annual reduction; RR per calendar year 0.946; 95% CI 0.938, 0.954) (ESM Table 3). Likewise, this rate decreased moderately but significantly in the population without diabetes, from 14.1 in 2009 to 13.0 in 2013 (2% annual decrease; RR per calendar year 0.98; 95% CI 0.967, 0.994). As a result, the RR, which compared people with and without diabetes, decreased significantly (interaction diabetes × calendar year: *p* < 0.001). Regarding time trend, the results were comparable in both sexes. The any amputation rate was more than twice as high in men as in women, with greater differences in the population with diabetes.

## Discussion

In this analysis over 5 years based on national health insurance data covering almost the entire population of Belgium, we found a substantial decline in the major amputation rate in people with diabetes, which was even more evident considering major amputation above the knee. In contrast, the major amputation rate remained unchanged in people without diabetes. Thus, the RR comparing people with and without diabetes decreased but remained high. A moderate decrease in minor LEAs was observed among people with and people without diabetes.

### Important differences

Although a number of studies have analysed amputation risk in people with diabetes, population-based and specifically nationwide studies analysing amputation risk in populations with and without diabetes are still limited. Studies differ significantly in terms of study design, as some studies counted every hospitalisation or every LEA, rather than just the first LEA in each year, as in our study. Some studies also only estimated crude incidence rates, which were usually considerably higher in the population with diabetes compared with our study, which estimated age-adjusted incidence rates [8]. Consequently, it is difficult to make correct comparisons between them. Only a few studies are partially comparable to our study in Belgium since they also counted one LEA per person [9–11, 14, 15]. These studies found LEA incidence rates among individuals with diabetes, which are well in line with our results (e.g. about 48 per 100,000 person-years in Finland in 2007 [15], about 36 per 100,000 person years in Italy in 2010 [14]; in our study between 30 and 42 per 100,000 person years).

Our finding concerning the time trend for major LEAs in the population with diabetes, with 8% reduction per calendar year, is in line with results from international studies, which mainly demonstrated a decrease in the incidence of major LEAs among people with diabetes [10, 14, 15, 17–19]. The annual reduction in these studies ranged from 5% [14] to 7% [17] per calendar year.

In contrast, epidemiological studies showed conflicting results regarding major amputation rates in the population without diabetes. In our study and in a previous German study [10] the risk of amputation among people without diabetes was stable. However, two other European studies reported a significant reduction in this rate [14, 15].

LEAs have a huge impact on the individual, their relatives and the community [1, 2, 4]. LEAs are related to an increased mortality risk of over 50% at 5 years. Higher age, more proximal amputations and presence of peripheral arterial disease, diabetes or renal insufficiency are factors that increase this mortality risk [1]. A substantial proportion of LEAs, particularly in people with diabetes, are preventable via the provision of appropriate healthcare [20]. Much effort has been made to reduce the amputation risk in the population with diabetes, e.g. the introduction of foot centres and cooperative care between general practitioners and diabetes specialists. Recent literature has not only shown relevant regional differences in major amputation rates [21], but also the inverse correlation of these rates with the provision of specialised diabetic foot care services [22].

Our data suggest that these efforts may have achieved positive effects, as the amputation risk in the population with diabetes decreased significantly, whereas it decreased to a less-clear extent in the population without diabetes. Nevertheless, when we consider the reduction in the major LEA amputation rate in the population with diabetes, it is still sixfold higher compared with people without diabetes. Hence, further efforts are needed to prevent diabetic foot ulcers.

### Strengths and weaknesses of the study

Several limitations have to be considered. First, the algorithm that was used to define diabetes status is based on reimbursed treatment, not on diagnosis. A careful assumption was made, based on inclusion in a diabetes care system and/or medical treatment with diabetes-specific medication and/or registration of repeated measurements of HbA_1c_. Inclusion in a diabetes care system is a clear indicator for a diagnosis of diabetes. Diabetes-related medical treatment is somewhat less precise in the case of metformin, as this can also be used for the treatment of obesity and polycystic ovary syndrome, although this is probably negligible in an older population. Repeated HbA_1c_ measurements suggest the presence of diabetes.

Second, as we used data from national health insurances, clinical data are unavailable, as are other variables such as socioeconomic status.

A key strength of our study is that we were able to analyse a nationwide dataset covering almost the entire Belgian population stratified by diabetes status and amputation level. Therefore, our study is—to our best knowledge—one of the few to report LEAs in a national population. Furthermore, our data on the number and type of LEAs are reliable as they are based on the same reimbursement data collected by the IMA/AIM. In addition, this study is based on a continuous 5 year observation period, and not on periodic sampling, in order to avoid methodological bias over time.

### Unanswered questions and future research

The major amputation rate in Belgium gradually declined during the study period in people with diabetes, which was particularly evident for major amputation above the knee. A weaker but also significant decrease was observed in minor amputation rate in people with, as well as those without, diabetes. In contrast, no change in any major LEA was found in individuals without diabetes. Despite all efforts to date, a large number of people still undergo major amputations. Considering the observed results, namely reductions in both major and minor amputation rates, to be a potential result of the implementation of specialised diabetic foot care services, nationwide coverage of such institutions and unrestricted access for individuals in need should be discussed as a primary future goal.

## Electronic supplementary material

ESM (PDF 190 kb)

## Data Availability

The data that support the findings of this study are available from IMA/AIM, but restrictions apply to the availability of these data, which were used under licence for the current study and so are not publicly available. Data are, however, available from the authors upon reasonable request and with permission of IMA/AIM.

## Abbreviations

ARd: Amputation rate for the estimated population with diabetes
ARn: Amputation rate for the population without diabetes
ESP: European Standard Population
IMA/AIM: InterMutualistisch Agentschap/Agence InterMutualiste
LEA: Lower-extremity amputations
