# Supplementary material for: Decreasing rates of major lower-extremity amputation in people with diabetes but not in those without: a nationwide study in Belgium
Source: Diabetologia. 2018 Jun 16;61(9):1966–77. doi: 10.1007/s00125-018-4655-6 (PMC6096627; doi:10.1007/s00125-018-4655-6)
Supplement: Supplementary file 1 — (PDF 190 kb) [file 125_2018_4655_MOESM1_ESM.pdf]

ESM Table 1: Major amputation rate, Belgium, 2009-2013 standardised to the European Standard Population 2013

	Ard[a]	Arn[a]	RR[b]
total population			
calendar year:			
2009	44.3	6.4	7
2010	45.4	6.3	7.2
2011	39.1	6.7	5.8
2012	35.9	6.3	5.7
2013	31.6	6.3	5
male population			
calendar year:			
2009	60.4	8.9	6.8
2010	61.9	8.6	7.2
2011	58.7	9.3	6.3
2012	53.6	8.8	6.1
2013	45.2	9	5
female population			
calendar year:			
2009	28.2	3.9	7.3
2010	28.8	4	7.2
2011	19.5	4.1	4.8
2012	18.3	3.7	4.9
2013	18.1	3.6	5

[a] Amputation rate per 100 000 person years in the population with diabetes (ARd) and without diabetes (ARn), standardized to the Belgian population 2011

[b] Relative risk comparing amputation rate in the population with and without diabetes (ARd/ARn)

ESM Table 2: Baseline characteristics of any amputations, Belgium, 2009-2013

	Diabetes				No diabetes				
	number of persons with amputation	mean age[a] (years)	number of total population	ARd[b]	number of persons with amputation	mean age* (years)	number of total population	ARn[b]	RR[c]
All amputations									
total population									
calendar year:									
2009	1691	71.0	678655	122.2	1278	68.9	10198663	14.1	8.6
2010	1710	71.1	738256	119.1	1229	68.8	10226043	13.5	8.8
2011	1674	70.5	793883	112.4	1253	68.0	10255320	13.8	8.2
2012	1735	70.9	846796	103.7	1181	68.2	10271802	13.0	8.0
2013	1774	71.0	896126	100.4	1193	68.2	10269852	13.0	7.7
male population									
calendar year:									
2009	1148	69.3	326803	181.3	736	66.0	5021923	18.6	9.8
2010	1121	69.5	354104	173.0	697	65.7	5038345	17.6	9.9
2011	1158	69.0	379168	172.7	716	66.0	5056551	18.1	9.6
2012	1191	69.6	402725	157.4	677	66.1	5067837	17.1	9.2
2013	1230	69.8	424291	152.0	683	66.0	5068800	17.0	8.9
female population									
calendar year:									
2009	543	74.7	351852	69.0	542	72.8	5176740	10.4	6.7
2010	589	74.3	384152	70.4	532	72.9	5187698	10.2	6.9
2011	516	73.8	414715	57.5	537	70.6	5198769	10.3	5.6
2012	544	73.8	444071	55.8	504	71.1	5203965	9.7	5.8
2013	544	73.8	471835	54.8	510	71.1	5201052	9.8	5.6

[a]Age at time of first major amputation

[b] Amputation rate per 100 000 person years in the population with diabetes (ARd) and without diabetes (ARn), standardized to the Belgian population 2011

[c] Relative risk comparing amputation rate in the population with and without diabetes (ARd/ARn)

[d] Estimated proportion of amputations attributable to diabetes among persons with diabetes

[e] Estimated proportion amputation risk attributable to diabetes among the entire population

ESM Table 3: Results of Poisson models: relative risks for any amputation, Belgium 2009-2013

Variables	Relative risk (95% CI)b		
	Total population	Men	Women
Model 1a (diabetes)			
Calendar year	0.946 (0.938,0.954)*	0.953 (0.944,0.962)*	0.932 (0.919,0.945)*
Male vs. female	2.383 (2.322,2.445)*	---------	---------
Age (years)[a]			
≥ 80	14.031 (12.216,16.116)*	8.678 (7.456,10.100)*	31.270 (24.331,40.188)*
70-79	10.817 (9.420,12.422)*	7.073 (6.083,8.224)*	22.795 (17.729,29.308)*
60-69	8.303 (7.228,9.538)*	5.746 (4.942,6.681)*	14.869 (11.546,19.147)*
50-59	6.070 (5.274,6.986)*	4.218 (3.620,4.914)*	10.72 (8.292,13.860)*
40-49	3.261 (2.801,3.797)*	2.152 (1.820,2.543)*	6.758 (5.154,8.861)*
Model 1b (no diabetes)			
Calendar year	0.980 (0.967,0.994)*	0.979 (0.963,0.996)*	0.982 (0.963,1.001)
Male vs. female	1.766 (1.698,1.836)*	---------	---------
Age (years)[a]			
≥ 80	47.432 (43.996,51.138)*	45.13 (41.005,49.67)*	46.387 (41.662,51.647)*
70-79	23.695 (21.913,25.621)*	26.287 (23.871,28.947)*	20.056 (17.874,22.505)*
60-69	11.348 (10.456,12.316)*	14.016 (12.701,15.467)*	7.710 (6.788,8.758)*
50-59	5.940 (5.447,6.478)*	6.769 (6.097,7.516)*	4.734 (4.145,5.408)*
40-49	2.374 (2.137,2.638)*	2.745 (2.423,3.109)*	1.823 (1.54,2.158)*
Model 2 (both diabetes and no diabetes combined)			
Calendar year	0.980 (0.964,0.996)*	0.979 (0.959,0.999)*	0.982 (0.960,1.004)
Diabetes (yes vs. no)	7.682 (7.283,8.104)*	8.997 (8.426,9.609)*	5.894 (5.461,6.362)*
Male vs. female	2.096 (2.033,2.165)*	---------	---------
Age (years)[a]			
≥ 80	32.278 (29.709,35.126)*	28.236 (25.472,31.370)*	36.248 (32.310,40.805)*
70-79	20.687 (19.035,22.517)*	20.111 (18.166,22.317)*	20.682 (18.384,23.342)*
60-69	13.505 (12.419,14.708)*	14.372 (12.985,15.945)*	10.616 (9.390,12.037)*
50-59	8.015 (7.345,8.758)*	8.603 (7.747,9.574)*	6.421 (5.638,7.328)*
40-49	3.105 (2.793,3.453)*	3.312 (2.919,3.759)*	2.692 (2.295,3.155)*
Diabetes x calendar year	0.965 (0.944,0.985)*	0.972 (0.947,0.998)*	0.949 (0.920,0.979)*

[a] Reference category: 18-49 years

* $p<0.05$

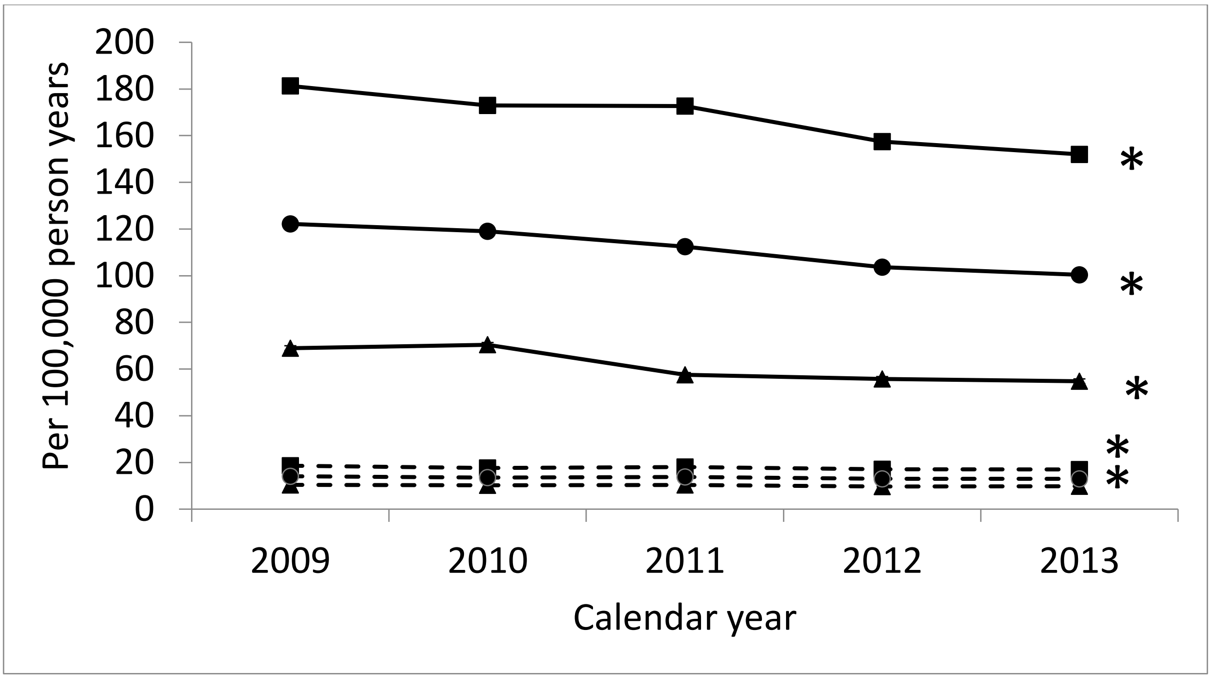

ESM Fig. 1 Time trend of age–sex standardised any amputation rate. Continuous lines = persons with diabetes; dotted lines = persons without diabetes; square = men; circle = men and women; triangle = women; * significant time trend (p value of Poisson model <0.05).
